# Entropy Production of Run-and-Tumble Particles

**DOI:** 10.3390/e26060443

**Published:** 2024-05-24

**Authors:** Matteo Paoluzzi, Andrea Puglisi, Luca Angelani

**Affiliations:** 1 Istituto per le Applicazioni del Calcolo, Consiglio Nazionale delle Ricerche, Via Pietro Castellino 111, I-80131 Napoli, Italy; matteo.paoluzzi@cnr.it; 2 Istituto dei Sistemi Complessi, Consiglio Nazionale delle Ricerche, Piazzale A. Moro 2, I-00185 Roma, Italy; andrea.puglisi@cnr.it; 3 Dipartimento di Fisica, Sapienza Università di Roma, Piazzale A. Moro 2, I-00185 Roma, Italy

**Keywords:** active matter, entropy production, non-equilibrium, exact results, run-and-tumble motion

## Abstract

We analyze the entropy production in run-and-tumble models. After presenting the general formalism in the framework of the Fokker–Planck equations in one space dimension, we derive some known exact results in simple physical situations (free run-and-tumble particles and harmonic confinement). We then extend the calculation to the case of anisotropic motion (different speeds and tumbling rates for right- and left-oriented particles), obtaining exact expressions of the entropy production rate. We conclude by discussing the general case of heterogeneous run-and-tumble motion described by space-dependent parameters and extending the analysis to the case of *d*-dimensional motions.

## 1. Introduction

Active matter is a recently established research field in statistical physics [1]. It includes systems made of (typically) many particles endowed with self-propulsion, the most prominent examples coming from biology, e.g., microswimmers or motile cells at the microscale [2] or birds and pedestrians at the macroscale [3], but encompasses also motile artificial particles at all scales [4]. Motility—which is a conversion of energy from some fuel/reservoir into the motion of each particle, is a fascinating ingredient for theoretical physics, as it implies a source of time-reversal symmetry breaking in the bulk of the systems [5,6,7,8], different from the usual force coming from the boundaries, which occurs in older examples of out-of-equilibrium systems such as fluids under the action of externally imposed gradients (e.g., heat flow, convection, turbulence, etc.) [9,10].

The interest of statistical physics in those systems is both at the level of a single active particle and at the level of large populations of active particles, since in both cases the lack of thermodynamic equilibrium triggers the appearance of unexpected phenomena [11,12,13,14]. A single self-propelling particle *hides* a complex arrangement of several internal degrees of freedom such as molecular motors actuating flagella, as in bacteria or sperms; it, therefore, may require nontrivial stochastic modeling, in contrast with passive Brownian particles [15]. A population of motile particles may exhibit collective behaviors that are not allowed when the motility ingredient is removed, typical examples being the polarization transition of aligning active particles [16] and the motility-induced phase separation for purely repulsive active particles [17,18].

One of the questions concerning the non-equilibrium statistical physics of the single active particle is how to characterize the dissipation occurring because of the time-reversal symmetry breaking induced by the self-propulsion mechanism [19]. A relevant approach to this problem is given by stochastic thermodynamics, which equips the theory of stochastic processes with a mesoscopic (fluctuating) definition of work, heat, and entropy production, including a fluctuating version of the second principle of thermodynamics [20,21,22]. The application of stochastic thermodynamics to single active particles has been developed in recent years, starting from models with continuous noise [23,24,25,26,27] such as active Ornstein–Uhlenbeck particles (AOUPs) and active Brownian particles (ABPs), and only more recently, it has been addressed also for time-discontinuous models such as run-and-tumble (RT) particles [28,29,30]. Such a model is considered a better description of certain biophysical systems, for instance, the *E. coli* bacteria which have reorientation dynamics dominated by sudden changes rather than rotational diffusion [31,32]. The less smooth mathematical structure of the model makes the problem interesting: for instance, ABPs and AOUPs have a finite entropy production even when translational thermal diffusion—often considered negligible in real applications—is set to zero in the model. On the contrary, an RT particle—under the influence of an external potential—in the limit of zero temperature becomes *strongly* time-irreversible, meaning that the time reversal of an observable trajectory in general is not observable, corresponding to an infinite entropy production [33]. The divergence is healed when a finite diffusivity D>0 is considered: typically—as seen also in this paper—the steady-state entropy production diverges for D→0. Morally, this corresponds to the fact that a model for active particles may have a finite rate for energy dissipation $\dot{W}$ even at zero temperature T=0, and therefore, it is not a paradox to find a divergence for the entropy production rate, expected on general grounds to be $\dot{W}/T$. A closer look at the problem, however, suggests that in many cases—particularly in biology—all energy conversion processes occurring inside an active particle are triggered by thermal processes (e.g., the dynamics of motor proteins is fueled by ATP molecules but the energy barriers among the protein configurations cannot be overcome at T=0), and therefore, one could expect $\dot{W}∼T$ so that one might obtain a finite entropy production rate in the limit T→0. This problem is, however, not the scope of this paper, and the question will be addressed in future research. The entropy production for run-and-tumble particles confined to move into a one-dimensional box was the subject of [28], following the recipe given in [34]. Here, we revisit this problem with a more straightforward derivation.

The structure of this paper is as follows. In Section 2, we review the minimal ingredients for the definition of entropy production of Markov processes described by a Fokker–Planck equation. In Section 3, we discuss entropy production for RT particles in 1D, starting with some known results rederived more straightforwardly, i.e., free RT particles and then RT particles in a harmonic potential. In Section 4, we give the expression for anisotropic models, i.e., RT particles in 1D with different tumbling rates and/or different self-propulsion velocities in the two directions of motion. In Section 5, we give a more general treatment which includes several cases of practical interest, and in Section 6, we extend the calculation to the *d*-dimensional case. Section 7 is devoted to conclusions.

## 2. Theoretical Setup within the Fokker–Planck Equation

Here, we briefly recall the theoretical framework for the computation of the entropy production rate in stochastic processes governed by Fokker–Planck-like equations [21]. Denoting by S(t) the entropy of the system at time *t*, we can decompose the rate of change of the entropy into two terms, Π and Φ, as

(1) $$\dot{S}=\frac{dS}{dt}=\Pi -\Phi \;,$$

where Π is the entropy production due to irreversible processes inside the system and Φ is the entropy flux from the system to the environment. The entropy production Π is non-negative, while Φ can have either sign.

We consider a generic stochastic process describing a particle moving in a one-dimensional space. The probability density function (PDF) P(x,t) to find the particle at position *x* at time *t* obeys the following continuity equation:

(2) $$\partial_{t}P\left(x,t\right)=-\partial_{x}J\left(x,t\right)\;,$$

where J(x,t) is the current and $\partial_{t}$ and $\partial_{x}$ denote, respectively, the time and space derivatives. In the case of the Fokker–Planck equation, one has the following constitutive relation linking the current to the probability:

(3) $$J\left(x,t\right)=[\mu f\left(x\right)-D\partial_{x}]P\left(x,t\right)\;,$$

where *D* is the diffusion constant, f(x) a generic space-dependent mechanical force acting on the particle, and μ the particle mobility.

The Gibbs entropy S(t) of the distribution P(x,t) is defined as

(4) S(t)=−∫dxP(x,t)logP(x,t),

and the rate of the entropy change reads

(5) $$\begin{array}{cc} \dot{S}\left(t\right) & =-\int dx\;\dot{P}\left(x,t\right)\left[1+\log P(x,t)\right]\;,\\ \; & =\int dx\;\partial_{x}J\left(x,t\right)\left[1+\log P(x,t)\right]\;,\\ \; & =-\int dx\;J\left(x,t\right)\partial_{x}\log P\left(x,t\right)\;, \end{array}$$

where we have used the continuity Equation (2) and integration by parts assuming vanishing distributions at boundaries. By using the relation (3), we can write

(6) $$\frac{J(x,t)}{DP(x,t)}=\frac{\mu f(x)}{D}-\partial_{x}\log P\left(x,t\right)\;,$$

and thus the expression for $\dot{S}\left(t\right)$ becomes

(7) $$\dot{S}=-\int dx\;\frac{J(x,t)}{D}\left[\mu f\left(x\right)-\frac{J(x,t)}{P(x,t)}\right]\;.$$

We finally obtain the following forms of the entropy rates defined in (1):

(8) $$\begin{array}{cc} \dot{S}\left(t\right) & =\Pi (t)-\Phi (t)\;, \end{array}$$

(9) $$\begin{array}{cc} \Pi (t) & =\int dx\;\frac{J^{2}\left(x,t\right)}{DP(x,t)}\;, \end{array}$$

(10) $$\begin{array}{cc} \Phi (t) & =\;\frac{\mu }{D}\int dx\;J\left(x,t\right)f\left(x\right)\;. \end{array}$$

As a functional of *J*, we immediately realize that Π(t) is non-negative, the integrand being proportional to $J^{2}$ with positive coefficients, while Φ can be either negative or positive. Π is the entropy production rate that can be also computed through the Kullback–Leibler divergence between the probability of a path of the system with respect to the time-reversal one.

In the stationary regime, we can compute the entropy production rate Π by noting that the rate of entropy change $\dot{S}$ must be zero,

(11) $$\begin{array}{c} {\dot{S}}_{st}=0=\Pi_{st}-\Phi_{st}\;, \end{array}$$

and, thus, we can compute Π through the expression for Φ since they are equal on stationary trajectories

(12) $$\begin{array}{c} \Pi_{st}=\Phi_{st}\;. \end{array}$$

When the Brownian particle reaches equilibrium, as, for example, in the presence of a confining potential V(x), the entropy production rate is zero

(13) $$\Pi_{st}=\frac{\mu }{D}\int dx\;f\left(x\right)\left[\mu f\left(x\right)-D\partial_{x}\right]P_{eq}\left(x\right)=0\;,$$

as is immediately clear considering that $f\left(x\right)=-\partial_{x}V\left(x\right)$ and $P_{eq}\left(x\right)\propto e^{-\mu V(x)/D}$. Instead, in the case of a driven Brownian particle, we have a finite entropy production. Indeed, in this case, the constant force produces a drift velocity v=μf, thus resulting in

(14) $$\Pi_{st}=\frac{v^{2}}{D}\;,$$

as obtained from (3), (10), and (12).

## 3. Run-and-Tumble Motion

We now calculate the entropy production in the case of run-and-tumble motions in the presence of thermal noise. We consider a particle that alternates sequences of *run* motion and *tumble* events: it moves at constant speed *v* in a given direction until it tumbles at rate α, randomly choosing the new direction of motion [35,36]. In the one-dimensional case analyzed here, there are only two possible directions of motion, let us say right and left (in the last Section 6, we generalize the analysis to higher dimensions). We assume that the particle is also subject to thermal noise, described by a diffusion coefficient *D*. We first treat the case of a free particle and then the motion in a confining harmonic potential. We derive in a simple way the exact expressions of the entropy production rates, without resorting to the explicit solutions of the kinetic equations of motion, reproducing the exact results known in the literature [28,29,30,37]. Unlike the previous section, for the sake of simplicity, here and in the following, we will omit in the reported equations the explicit dependence on the *x* and *t* variables of the various quantities.

### 3.1. Free Run-and-Tumble Particles

We first analyze the case of a free run-and-tumble particle. We indicate with R(x,t) the probability density function to find the particle at position *x* at the time *t* moving towards the right, and with L(x,t) the probability density function for the particle moving towards the left. The coupled kinetic equations describing the run-and-tumble motion in the presence of thermal noise are

(15) $$\begin{array}{cc} \partial_{t}R & =D\partial_{x}^{2}R-v\partial_{x}R+\frac{\alpha }{2}\left(L-R\right)\;, \end{array}$$

(16) $$\begin{array}{cc} \partial_{t}L & =D\partial_{x}^{2}L+v\partial_{x}L-\frac{\alpha }{2}\left(L-R\right)\;. \end{array}$$

Once we introduce the currents

(17) $$\begin{array}{cc} J_{R} & =vR-D\partial_{x}R\;, \end{array}$$

(18) $$\begin{array}{cc} J_{L} & =-vL-D\partial_{x}L\;, \end{array}$$

(19) $$\begin{array}{cc} J_{LR} & =\frac{\alpha }{2}\left(R-L\right)\;, \end{array}$$

we can write the equations of motion as follows:

(20) $$\begin{array}{cc} \partial_{t}R & =-\partial_{x}J_{R}-J_{LR}\;, \end{array}$$

(21) $$\begin{array}{cc} \partial_{t}L & =-\partial_{x}J_{L}+J_{LR}\;. \end{array}$$

The entropy *S* is given by the sum of the two entropies

(22) $$\begin{array}{cc} S & =S_{R}+S_{L}\;, \end{array}$$

(23) $$\begin{array}{cc} S_{R} & =-\int dx\;R\log R\;, \end{array}$$

(24) $$\begin{array}{cc} S_{L} & =-\int dx\;L\log L\;. \end{array}$$

Performing the time derivative, we have

(25) $$\begin{array}{cc} \dot{S} & ={\dot{S}}_{R}+{\dot{S}}_{L}\;, \end{array}$$

(26) $$\begin{array}{cc} {\dot{S}}_{R} & =-\int dx\;\partial_{t}R\left(1+\log R\right)\;, \end{array}$$

(27) $$\begin{array}{cc} {\dot{S}}_{L} & =-\int dx\;\partial_{t}L\left(1+\log L\right)\;, \end{array}$$

and, using Equations (20) and (21), we obtain

(28) $$\begin{array}{cc} {\dot{S}}_{R} & =\int dx\;\left(\partial_{x}J_{R}+J_{LR}\right)\left(1+\log R\right)\\ \; & =-\int dx\;\frac{J_{R}}{R}\partial_{x}R+\int dx\;J_{LR}\left(1+\log R\right)\;. \end{array}$$

and similarly

(29) $$\begin{array}{cc} {\dot{S}}_{L} & =\int dx\;\left(\partial_{x}J_{L}-J_{LR}\right)\left(1+\log L\right)\\ \; & =-\int dx\;\frac{J_{L}}{L}\partial_{x}L-\int dx\;J_{LR}\left(1+\log L\right)\;, \end{array}$$

having considered that distributions vanish at infinity. Using the expressions for $J_{R,L}$, we can write

(30) $$\begin{array}{cc} \frac{\partial_{x}R}{R} & =\frac{1}{D}\left(v-\frac{J_{R}}{R}\right)\;, \end{array}$$

(31) $$\begin{array}{cc} \frac{\partial_{x}L}{L} & =-\frac{1}{D}\left(v+\frac{J_{L}}{L}\right)\;, \end{array}$$

so that, upon neglecting boundary terms, we obtain

(32) $$\begin{array}{cc} \dot{S} & =\Pi -\Phi \;, \end{array}$$

(33) $$\begin{array}{cc} \Pi & =\frac{1}{D}\int dx\;\left(\frac{J_{R}^{2}}{R}+\frac{J_{L}^{2}}{L}\right)+\frac{\alpha }{2}\int dx\;\left(R-L\right)\log\frac{R}{L}\;, \end{array}$$

(34) $$\begin{array}{cc} \Phi & =\frac{v}{D}\int dx\;\left(J_{R}-J_{L}\right)\;. \end{array}$$

At the steady state, we obtain $\dot{S}=0$ so that $\Pi_{st}=\Phi_{st}$, and thus, the entropy production rate is given by

(35) $$\begin{array}{c} \Pi_{st}=\frac{v}{D}\int dx\;\left(J_{R}-J_{L}\right)\;. \end{array}$$

Once we introduce

(36) $$\begin{array}{c} P\equiv R+L\;, \end{array}$$

(37) $$\begin{array}{c} Q\equiv R-L\;, \end{array}$$

with ∫dxP(x)=1, we obtain

(38) $$\begin{array}{c} J_{R}-J_{L}=vP-D\partial_{x}Q\;, \end{array}$$

so that

(39) $$\begin{array}{c} \Pi_{st}=\frac{v^{2}}{D}\;. \end{array}$$

We note that the above result is the same as the one obtained for a driven Brownian particle. Indeed, we observe that a free run-and-tumble particle with diffusion can be viewed as a drift-diffusive particle going constantly in the direction parallel to its own driving force, even if such a force (proportional to the velocity of the particle) changes direction at random times. The process of tumbling is instantaneous and therefore does not add any contribution to the entropy production.

### 3.2. Run-and-Tumble Particles in Harmonic Potential

We now consider the case of a run-and-tumble particle in a confining (harmonic) potential

(40) $$V\left(x\right)=\frac{k}{2}x^{2},$$

where *k* is the potential stiffness. The Fokker–Planck equations for *R* and *L* are

(41) $$\begin{array}{cc} \partial_{t}R & =-\partial_{x}J_{R}-J_{LR}\;, \end{array}$$

(42) $$\begin{array}{cc} \partial_{t}L & =-\partial_{x}J_{L}+J_{LR}\;, \end{array}$$

where

(43) $$\begin{array}{cc} J_{R} & =(v+\mu f-D\partial_{x})R\;, \end{array}$$

(44) $$\begin{array}{cc} J_{L} & =(-v+\mu f-D\partial_{x})L\;, \end{array}$$

(45) $$\begin{array}{cc} J_{LR} & =\frac{\alpha }{2}\left(R-L\right)\;, \end{array}$$

and $f\left(x\right)=-\partial_{x}V\left(x\right)=-kx$ is the force field. Proceeding as before, we can write the entropy rate as

(46) $$\begin{array}{cc} \dot{S} & =\Pi -\Phi \;, \end{array}$$

(47) $$\begin{array}{cc} \Pi & =\frac{1}{D}\int dx\;\left(\frac{J_{R}^{2}}{R}+\frac{J_{L}^{2}}{L}\right)+\frac{\alpha }{2}\int dx\;\left(R-L\right)\log\frac{R}{L}\;, \end{array}$$

(48) $$\begin{array}{cc} \Phi & =\frac{v}{D}\int dx\;\left(J_{R}-J_{L}\right)-\frac{\mu k}{D}\int dx\;x\left(J_{R}+J_{L}\right)\;. \end{array}$$

In the steady state, we have $\Pi_{st}=\Phi_{st}$ and, considering that $J=J_{R}+J_{L}=0$, we obtain

(49) $$\Pi_{st}=\frac{v}{D}\int dx\;\left(J_{R}-J_{L}\right)\;.$$

By noting that

(50) $$J_{R}-J_{L}=vP-\mu kxQ-D\partial_{x}Q\;,$$

where *P* and *Q* are defined in (36)–(37), we have (considering the normalization condition and the vanishing of the distributions at infinity)

(51) $$\Pi_{st}=\frac{v}{D}\left(v+I\right)\;,$$

where

(52) I≡−μk∫dxxQ,

From (41), (42), and (45), in the stationary regime, we have

(53) $$\partial_{x}\left(J_{R}-J_{L}\right)=-\alpha Q\;,$$

and multiplying by the force and integrating over space gives

(54) $$\mu k\int dx\;x\partial_{x}\left(J_{R}-J_{L}\right)=\alpha I\;.$$

Integrating by parts, we obtain

(55) $$\alpha I=-\mu k\int dx\left(J_{R}-J_{L}\right)=-\mu k\left(v+I\right)\;,$$

and then

(56) $$I=-\frac{\mu kv}{\alpha +\mu k}\;.$$

Substituting in (51), we finally obtain the expression of the entropy production rate:

(57) $$\Pi_{st}=\frac{v^{2}}{D}\;\frac{\alpha }{\alpha +\mu k}\;.$$

The above expression is in agreement with that reported in [30]—see Equation (55)—and also in [37], Equation (41), where it was obtained using a path integral approach. We note that for k=0, we recover the previous expression (39), valid for a free run-and-tumble particle. It is remarkable that the above result has been obtained without resorting to the exact stationary solution of the run-and-tumble equations, which indeed in this case cannot be written in closed form [38].

## 4. Anisotropic Run-and-Tumble Motion

We extend here the analysis of the previous section to the case of particles performing anisotropic run-and-tumble motion, i.e., we consider tumbling rates and speeds which depend on the orientation of the particle, $\alpha_{R}\neq \alpha_{L}$ and $v_{R}\neq v_{L}$. These parameters are assumed to be constant in time and space, which will allow us to obtain exact results for the entropy production. In the next section, we relax the spatial homogeneity condition, allowing the speeds and tumbling rates to depend explicitly on the variable *x*. We treat here the case of motion in the presence of a harmonic potential $V\left(x\right)=\frac{k}{2}x^{2}$, the free case being recovered in the limit of zero spring constant, k=0. The Fokker–Planck equations for *R* and *L* are

(58) $$\begin{array}{cc} \partial_{t}R & =-\partial_{x}J_{R}-J_{LR}\;, \end{array}$$

(59) $$\begin{array}{cc} \partial_{t}L & =-\partial_{x}J_{L}+J_{LR}\;, \end{array}$$

where

(60) $$\begin{array}{cc} J_{R} & =(v_{R}+\mu f-D\partial_{x})R\;, \end{array}$$

(61) $$\begin{array}{cc} J_{L} & =(-v_{L}+\mu f-D\partial_{x})L\;, \end{array}$$

(62) $$\begin{array}{cc} J_{LR} & =\frac{1}{2}\left(\alpha_{R}R-\alpha_{L}L\right)\;, \end{array}$$

and $f\left(x\right)=-\partial_{x}V\left(x\right)=-kx$ is the force field. The entropy rate is

(63) $$\begin{array}{cc} \dot{S} & =\Pi -\Phi \;, \end{array}$$

(64) $$\begin{array}{cc} \Pi & =\frac{1}{D}\int dx\;\left(\frac{J_{R}^{2}}{R}+\frac{J_{L}^{2}}{L}\right)+\frac{1}{2}\int dx\;\left(\alpha_{R}R-\alpha_{L}L\right)\log\frac{R}{L}\;, \end{array}$$

(65) $$\begin{array}{cc} \Phi & =\frac{1}{D}\int dx\;\left(v_{R}J_{R}-v_{L}J_{L}\right)-\frac{\mu k}{D}\int dx\;x\left(J_{R}+J_{L}\right)\;. \end{array}$$

In the steady state, we have

(66) $$\Pi_{st}=\frac{1}{D}\int dx\;\left(v_{R}J_{R}-v_{L}J_{L}\right)\;.$$

By using (60) and (61), we have

(67) $$\begin{array}{ccc} D\Pi_{st} & = & v_{R}^{2}\int dxR+v_{L}^{2}\int dxL\\ & + & \mu kv_{L}\int dxxL-\mu kv_{R}\int dxxR\;. \end{array}$$

We now observe that the first two integrals in (67) are given by

(68) $$\begin{array}{cc} \int dxR & =\frac{\alpha_{L}}{\alpha_{R}+\alpha_{L}}\;, \end{array}$$

(69) $$\begin{array}{cc} \int dxL & =\frac{\alpha_{R}}{\alpha_{R}+\alpha_{L}}\;, \end{array}$$

as obtained considering the normalization condition of P=R+L and that the integral of $J_{LR}$ (62) must be zero, as follows from Fokker–Planck equations in the stationary regime.

Now we consider the case k>0. From (58) and (59) in the stationary regime, we have

(70) $$\partial_{x}\left(v_{R}J_{R}-v_{L}J_{L}\right)=-\frac{v_{R}+v_{L}}{2}\left(\alpha_{R}R-\alpha_{L}L\right)\;,$$

and then, multiplying by μkx and integrating over *x*

(71) $$\mu k\int dx\;x\partial_{x}\left(v_{R}J_{R}-v_{L}J_{L}\right)=\frac{v_{R}+v_{L}}{2}\left(\alpha_{R}I-\alpha_{L}Y\right)\;,$$

where

(72) $$\begin{array}{cc} I & \equiv -\mu k\int dx\;xR\;, \end{array}$$

(73) $$\begin{array}{cc} Y & \equiv -\mu k\int dx\;xL. \end{array}$$

Integrating by parts, we obtain

(74) $$\mu k\int dx\;\left(v_{R}J_{R}-v_{L}J_{L}\right)=-\frac{v_{R}+v_{L}}{2}\left(\alpha_{R}I-\alpha_{L}Y\right)\;.$$

The quantities *I* and *Y* are related to each other. Indeed, in the steady state, the total current is zero and then, using (60) and (61), we have

(75) $$0=\int dx\left(J_{R}+J_{L}\right)=v_{R}\int dxR-v_{L}\int dxL+I+Y\;.$$

Using (68) and (69), we obtain

(76) $$I+Y=\frac{v_{L}\alpha_{R}-v_{R}\alpha_{L}}{\alpha_{R}+\alpha_{L}}\;.$$

Combining Equations (74) and (76)—together with (68) and (69)—we obtain an equation for *I*, whose solution is

(77) $$I=\frac{\alpha_{L}}{\alpha_{R}+\alpha_{L}}\;\frac{\alpha_{R}v_{L}-\alpha_{L}v_{R}-2\mu kv_{R}}{2\mu k+\alpha_{R}+\alpha_{L}}\;.$$

Using (76), we obtain for *Y*

(78) $$Y=\frac{\alpha_{R}}{\alpha_{R}+\alpha_{L}}\;\frac{\alpha_{R}v_{L}-\alpha_{L}v_{R}+2\mu kv_{L}}{2\mu k+\alpha_{R}+\alpha_{L}}\;.$$

Substituting in (74) and using (66), we finally arrive at the expression of the entropy production rate for k>0:

(79) $$\Pi_{st}=\frac{{\left(v_{R}+v_{L}\right)}^{2}}{D}\;\frac{\alpha_{R}\alpha_{L}}{\left(2\mu k+\alpha_{R}+\alpha_{L}\right)\left(\alpha_{R}+\alpha_{L}\right)}\;.$$

Defining the average speed $v=(v_{R}+v_{L})/2$, the average tumbling rate $\alpha =(\alpha_{R}+\alpha_{L})/2$, and the tumbling rate semidifference $\delta =(\alpha_{R}-\alpha_{L})/2$, the EPR takes the simple form

(80) $$\Pi_{st}=\frac{v^{2}}{D}\;\frac{\alpha^{2}-\delta^{2}}{\alpha (\mu k+\alpha )}\;.$$

For $\alpha_{R}=\alpha_{L}$, i.e., δ=0, the EPR reads

(81) $$\Pi_{st}=\frac{v^{2}}{D}\;\frac{\alpha }{\mu k+\alpha },\;\delta =0\;,$$

similar to the expression obtained in the isotropic case (57) with the average speed $v=(v_{R}+v_{L})/2$.

In the free case, the EPR can be computed directly by putting k=0 into Equation (67), which—together with Equations (68) and (69)—leads to

(82) $$\Pi_{st}=\frac{1}{D}\frac{\alpha_{L}v_{R}^{2}+\alpha_{R}v_{L}^{2}}{\alpha_{R}+\alpha_{L}}\;.$$

We first note that the limit k→0 of Equation (79) is different from (82), i.e., it is singular. This has already been noticed, in the case $v_{R}=v_{L}$, in [39]. The reason is that in the free anisotropic case, a residual total current is present even in the steady state (i.e., asymptotically in time) and that is an additional source of dissipation. Such a current vanishes as soon as k>0, even very small. Formula (82) gives for $\alpha_{R}=\alpha_{L}=\alpha$:

(83) $$\Pi_{st}=\frac{1}{D}\frac{v_{R}^{2}+v_{L}^{2}}{2}\;,$$

a result already obtained in [29] by means of a trajectory-based approach. When $v_{R}\;=\;v_{L}\;=\;v$, we instead obtain [39]

(84) $$\Pi_{st}=\frac{v^{2}}{D}\;,$$

i.e., the same result for the free isotropic case, remarkably independent from the tumbling rates.

**Figure 1:**
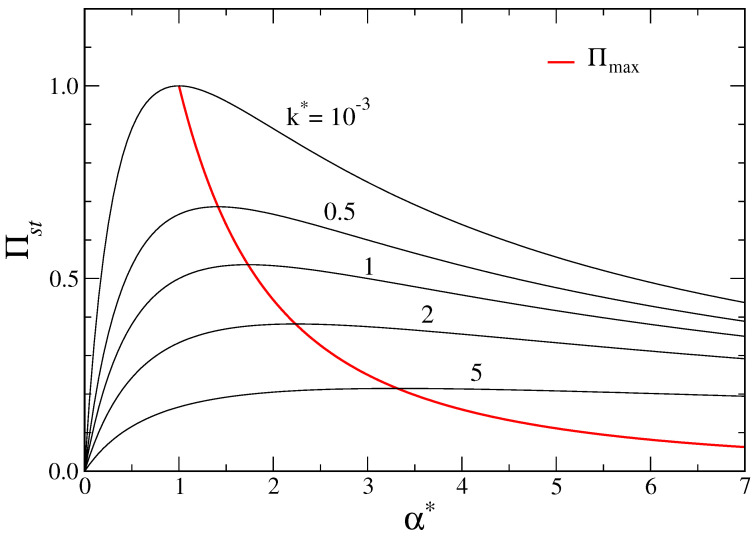
Entropy production rate at stationarity —see (79) in the text—as a function of the relative tumbling rate $\alpha^{*}=\alpha_{R}/\alpha_{L}$ for different values of the reduced harmonic constant $k^{*}=k/\alpha_{L}=10^{-3},0.5,1,2,5$. The red line is the maximum EPR $\Pi_{max}$ vs. $\alpha_{max}^{*}\left(k^{*}\right)$. Units are such that v=1, D=1, μ=1.

It is worth noting that in the general case, for a fixed external potential (k>0), the EPR reaches its maximum value $v^{2}/D$ in the symmetric case (δ=0) and for large tumbling rates (α→∞). However, some interesting behaviors of the EPR are obtained by considering some parameters fixed. While it is true that by fixing *k* and α, the maximum EPR $v^{2}{\left(1+\mu k/\alpha \right)}^{-1}/D$ is always obtained for δ=0, in the case of fixed *k* and $\alpha_{L}$, one has that the maximum EPR is reached for $\alpha^{*}=\alpha_{R}/\alpha_{L}>1$ (see Figure 1). The same would happen by fixing the value of $\alpha_{R}$, with the relative tumbling rate given by $\alpha^{*}=\alpha_{L}/\alpha_{R}$.

## 5. General Run-and-Tumble Motion

Let us now treat the very general case of anisotropic and heterogeneous run-and-tumble motion. We consider the possibility that tumbling rates and speeds not only could be different for left- and right-oriented particles but also could depend on the spatial variables $\alpha_{R,L}\left(x\right)$ and $v_{R,L}\left(x\right)$. Moreover, we consider the presence of a generic external force f(x), not necessarily originated by a confining quadratic potential. In this general case, the Fokker–Planck equations for *R* and *L* can be written as follows (for the sake of simplicity we omit the dependence on the *x*-variable of the physical parameters):

(85) $$\begin{array}{cc} \partial_{t}R & =-\partial_{x}J_{R}-J_{LR}\;, \end{array}$$

(86) $$\begin{array}{cc} \partial_{t}L & =-\partial_{x}J_{L}+J_{LR}\;, \end{array}$$

where

(87) $$\begin{array}{cc} J_{R} & =(v_{R}+\mu f-D\partial_{x})R\;, \end{array}$$

(88) $$\begin{array}{cc} J_{L} & =(-v_{L}+\mu f-D\partial_{x})L\;, \end{array}$$

(89) $$\begin{array}{cc} J_{LR} & =\frac{1}{2}\left(\alpha_{R}R-\alpha_{L}L\right)\;. \end{array}$$

The entropy rate is

(90) $$\begin{array}{cc} \dot{S} & =\Pi -\Phi \;, \end{array}$$

(91) $$\begin{array}{cc} \Pi & =\frac{1}{D}\int dx\;\left(\frac{J_{R}^{2}}{R}+\frac{J_{L}^{2}}{L}\right)+\frac{1}{2}\int dx\;\left(\alpha_{R}R-\alpha_{L}L\right)\log\frac{R}{L}\;, \end{array}$$

(92) $$\begin{array}{cc} \Phi & =\frac{1}{D}\int dx\;\left(v_{R}J_{R}-v_{L}J_{L}\right)+\frac{\mu }{D}\int dx\;f\left(J_{R}+J_{L}\right)\;. \end{array}$$

In the steady state, we have

(93) $$\Pi_{st}=\frac{1}{D}\int dx\;\left(v_{R}J_{R}-v_{L}J_{L}\right)+\frac{\mu }{D}\int dx\;f\left(J_{R}+J_{L}\right)\;.$$

In the case of vanishing flows at steady state $J_{R}+J_{L}=0$ (as occurs, for example, in the presence of confining potentials), the above expression is formally identical to the one obtained in the previous section (66), but now the parameters $v_{R,L}$ and $\alpha_{R,L}$ are explicitly space-dependent quantities. In the general case, it is not possible to obtain exact expressions of the EPR, and we need to resort to a numerical solution of kinetic equations or numerical simulations of the trajectories of the run-and-tumble particles.

We conclude this section by mentioning some particular case studies, which are interesting for their physical or biological relevance.

**Photokinetic bacteria.** Photokinetic bacteria are characterized by spatially varying speed which depends on local light intensity *I* [40]. For static nonhomogeneous light fields I(x), we can describe the particle dynamics through a space-dependent speed v(x) [41] (we assume equal left and right speeds)
  (94) v(x)=v(I(x)).
  **Chemotaxis.** In the presence of nutrient concentration, some motile bacteria modify their tumble rates to effectively direct their movement toward the food source [31,35]. We can describe such a phenomenon by expressing the tumble rates in terms of the chemotactic field c(x). In the limit of a weak concentration gradient, we can write [35,42,43]
  (95) $$\begin{array}{ccc} \alpha_{R}\left(x\right) & = & \alpha -\gamma v\partial_{x}c\left(x\right)\;, \end{array}$$
  (96) $$\begin{array}{ccc} \alpha_{L}\left(x\right) & = & \alpha +\gamma v\partial_{x}c\left(x\right)\;, \end{array}$$
  with γ measuring the strength of the particle reaction to chemical gradients, and we have assumed equal speeds $v_{R}=v_{L}=v$. Moreover, it is interesting to consider more realistic models of bacterial dynamics including noninstantaneous tumbling, with the addition of finite dwell times in the tumble state and possibly different rates of transition between the run and tumble states [44,45].
**Generic confining potentials.** In the previous sections, we analyzed the case of a force field $f\left(x\right)=-\partial_{x}V\left(x\right)$ originated by quadratic potentials $V\left(x\right)\propto x^{2}$. It would be interesting to consider the generic confining potential [46,47]
  (97) $$V\left(x\right)={a|x|}^{p}\;,\;p\geq 1\;,$$
  and investigate the dependence on the exponent *p*. Furthermore, of interest is the case of double-well potentials
  (98) $$V\left(x\right)=ax^{4}-bx^{2}+cx\;,$$
  in its symmetric (c=0) or asymmetric (c≠0) version.
**Ratchet potentials.** Finally, we mention the study of the ratchet effect [5]. In this case, the active motion takes place in the presence of a periodic asymmetric potential, giving rise to unidirectional motion with a stationary flow of particles, $J_{R}+J_{L}\neq 0$. In the case of a piecewise-linear ratchet potential, the entropy production for particles with equal tumbling rates and speeds was analyzed in [48].

## 6. Run-and-Tumble Motion in $R^{d}$

To date, we have considered the case of one-dimensional motions. Here, we extend the analysis to *d*-dimensional run-and-tumble walks. We consider a particle that, in the free case, moves along straight lines with velocity v=ve, where *v* is the speed and e a unit vector in $R^{d}$, and randomly changes its direction of motion e with rate α, choosing the new direction from a uniform distribution. We first derive the general expression of the EPR considering generic space- and orientation-dependent speed and tumbling rate, v(x,e) and α(x,e). Then we specialize to the simple case of constant *v* and α, showing the exact expression of the EPR in the presence of a harmonic potential.

By denoting with p(x,t;e) the PDF to find the particle at position $x\in R^{d}$ at time *t* with velocity orientation e, the kinetic equation of the run-and-tumble motion can be written as [49]

(99) $$\partial_{t}p=-\nabla \cdot j+\alpha \left(P-1\right)p\;,$$

where the current j is as follows (we consider the presence of thermal noise and generic force field f(x)):

(100) $$j=\left(-D\nabla +ve+\mu f\right)p\;,$$

and we introduced the projector operator

(101) $$Pp\left(x,t;e\right)=\int \frac{de}{\Omega_{d}}\;p\left(x,t;e\right)\;,$$

with $\Omega_{d}\;=\;2\pi^{d/2}/\Gamma \left(d/2\right)$ the solid angle in *d*-dimension. Hereafter, we consider normalization condition ∫dxdep(x,t;e)=1. We define the total entropy *S* as, generalizing (22),

(102) S(t)=∫des(t;e),

where the orientation-dependent entropy *s* is

(103) s(t;e)=−∫dxp(x,t;e)logp(x,t;e).

By performing a derivation similar to that of the previous section, we arrive at the expression of the entropy rate

(104) $$\begin{array}{cc} \dot{S} & =\Pi -\Phi \;, \end{array}$$

(105) $$\begin{array}{cc} \Pi & =\int dx\int de\;\left[\frac{{\left|j\right|}^{2}}{Dp}+\alpha \left(p-Pp\right)\log p\right]\;, \end{array}$$

(106) $$\begin{array}{cc} \Phi & =\frac{1}{D}\int dx\int de\;\left(ve+\mu f\right)\cdot j\;, \end{array}$$

which generalize to dimension d>1 the expressions previously obtained in (91), (92). In the steady state, we have Π=Φ, and, assuming a null net current ∫dej=0, we have that the EPR reads

(107) $$\Pi_{st}=\frac{1}{D}\int dx\int de\;ve\cdot j\;.$$

The results obtained so far are valid in the general, nonhomogeneous, and nonisotropic case, i.e., for generic v(x,e) and α(x,e). We now specify the calculation to the case of constant parameters *v* and α, extending the analysis of planar motions in [30] to $R^{d}$ with generic d>1. By using (100), we can write the EPR as

(108) $$\Pi_{st}=\frac{v^{2}}{D}\left[1+\frac{\mu }{v}\int dx\int de\;p\;e\cdot f\right]\;,$$

having used the normalization condition and neglecting boundary terms. Consider below a force field due to a harmonic potential, i.e., f=−kx. By substituting (100) in (99) in the stationary regime, multiplying by e·x, integrating over dx and de, and using integration by parts, we arrive at an equation for the quantity

(109) I≡∫dx∫depe·x,

appearing in the second term of (108), which is

(110) (α−dkμ)I=v−kμ(1+d)I,

leading to

(111) $$I=\frac{v}{\alpha +\mu k}\;.$$

Substituting in (108), we finally obtain the expression of the EPR

(112) $$\Pi_{st}=\frac{v^{2}}{D}\frac{\alpha }{\alpha +\mu k}\;,$$

which is the same as that obtained in the one-dimensional case (57) and is therefore independent of spatial dimensions.

## 7. Conclusions

We computed the average entropy production rate in the steady state for a noninteracting run-and-tumble particle in several different physical setups. The general strategy is to start from the kinetic equations and then compute the entropy flux, identical to the entropy production in a steady state. The entropy flux—in the absence of a total net current (e.g., in confined or spatially symmetric situations)—is seen to be proportional to the difference of left–right currents $J_{L},J_{R}$, weighted by the left–right speeds $v_{L},v_{R}$ (Equations (35), (49), and (66) in the different situations). The left–right currents endow also a dependence upon the tumbling rates. Such a weighted difference can be computed, in most of the considered situations, *without* computing the single currents but going directly to compute their weighted difference. This is a shortcut which allows us to revisit the free and harmonically confined cases, which already had a solution in the literature. The power of the method enables us to compute the entropy production rate also in nonsymmetric setups where the tumbling rates and the velocities are different when particles go to the left or to the right. A discussion of the more general case where all parameters are space-dependent was also presented, but explicit results cannot be usually obtained: a few cases of physical relevance are discussed with some detail. Finally, we extended the calculation to the case of run-and-tumble motions in a *d*-dimensional space, showing the formal expression of the EPR in the general case of space- and orientation-dependent parameters and reporting the exact solution in the case of harmonic potential and constant speed and tumbling rate. Future research should focus on the entropy production for interacting RT systems exhibiting motility-induced phase separation [18], where non-equilibrium density fluctuations have been investigated, usually starting from opportune coarse-graining descriptions [50,51,52]. Finally, the theoretical framework considered here might be tested against experiments such as the ones recently performed on different biological systems, where EPR can be computed in a model-independent fashion [53,54,55].

## Data Availability

Data is contained within the article.
